# Autophagy protein LC3 regulates the fibrosis of hypertrophic scar by controlling Bcl-xL in dermal fibroblasts

**DOI:** 10.18632/oncotarget.20771

**Published:** 2017-09-08

**Authors:** Jihong Shi, Shan Shi, Bin Wu, Jian Zhang, Yan Li, Xue Wu, Julei Zhang, Kejia Wang, Bin Zhao, Weixia Cai, Xiaozhi Bai, Dahai Hu, Hao Guan

**Affiliations:** ^1^ Department of Burns and Cutaneous Surgery, Xijing Hospital, Fourth Military Medical University, Xi’an, Shaanxi 710032, China; ^2^ Department of Burns and Plastic Surgery, Affiated Hospital of Yan’an University, Yan’an, Shaanxi 716000, China

**Keywords:** microtubule-associated protein 1 light chain 3 (LC3), hypertrophic scar, fibroblast, fibrosis, Bcl-xL

## Abstract

Hypertrophic scar (HS) is a serious skin fibrotic disease characterized by excessive hypercellularity and extracellular matrix (ECM) component deposition. Autophagy is a tightly regulated physiological process essential for cellular maintenance, differentiation, development and homeostasis. However, during the formation of HS, whether and how autophagy is regulated in dermal fibroblasts are still far from elucidated. Here we detected the autophagic capacity in HS and normal skin (NS) counterparts, explored and verified the key regulatory molecules of autophagy in HS-derived fibroblasts (HSFs), and validated the data using rabbit ear scar model. Transmission electron microscopy (TEM) and immunostaining data showed that LC3-positive cells and autophagosomes in HS/HSFs were more intensive relative to those in NS/NSFs groups. Knockdown of LC3 (shLC3) could significantly block the expressionof type I collagen (Col 1, p < 0.01) and type III collagen (Col 3, p < 0.01) and thus inhibit the fibrosis of HSFs. shLC3 resistant to autophagy was shown to be Bcl-xL-, not Bcl-2-dependent, and silencing of Bcl-xL (sibcl-xL) significantly increased apoptosis of HSFs (p < 0.01). Immunofluorescence results showed that instead of inhibiting α-SMA protein expression, shLC3 could change its architecture arrangement in HSFs. sibcl-xL showed that Bcl-xL was a key signaling molecule involved in HSFs autophagy. More importantly, both shLC3 and sibcl-xL obviously improved the appearance and architecture of the rabbit ear scar, and reduced scar formation on the rabbit ear. Therefore, the aberration of LC3 protein processing compromised autophagy in HS might associate with its pathogenesis in wound repair. LC3 regulated HS fibrosis by controlling the expression of Bcl-xL in HSFs. Thus, Bcl-xL might serve as a potential molecular target, providing a novel strategy for HS therapy.

## INTRODUCTION

Autophagy is used as a cellular response in which proteins, organelles, and portion of cytoplasm are engulfed, digested, and recycled to sustain cellular metabolism during stress [1, 2]. However, prolonged autophagy activation can also result in dysfunction of cellular organelles and even self-destruction of cells [3, 4]. This process is physiologically essential for the maintenance of cellular functions, cell viability, differentiation and development in mammals [5-8], and also serves as an adaptive mechanism to protect organisms against diverse pathological insults [5, 9, 10]. During autophagy, the cup-shaped pre-autophagosome engulfs cytosolic components to form an autophagosome, which subsequently fuses with a lysosome, leading to the proteolytic degradation of internal components of the autophagosome by lysosomal lytic enzymes [11, 12]. LC3, an intrinsic component of autophagosomal membranes, has been characterized as an autophagosomal marker during mammalian autophagy [13, 14].

Hypertrophic scar (HS), which is raised, red, inflexible, and responsible for serious functional and cosmetic problems, is pathologically a significant skin fibrotic disease and has negative impacts on patient appearance, skeletal muscular functions, and quality of life in general [15-19]. HS is characterized by excessive hypercellularity and extracellular matrix (ECM) deposition and its formation usually results from an abnormal processing of the tightly regulated tissue repair after traumatic injury to the skin. One major feature of HS is the disorder of metabolism for collagen-based ECM proteins [20-22], such as type I collagen (Col 1) and type III collagen (Col 3). Generally, HS formation is regarded as a consequence of the disruption of the homeostasis mechanism of skin. Many studies [5-10, 23-27] have shown that autophagy plays important roles for the pathogenesis of many human diseases such as tumorigenesis, neurodegenerative and neuromuscular diseases, aging, cardiomyopathies, bacterial and viral infections, which associate with homeostasis of tissue structures and functions. Existing studies [23-27] suggest that autophagy in skin dermis is associated with the maintenance, viability, differentiation and survival of fibroblasts during wound healing and repair, so as to lead to the pathogenesis of pathological scars, such as HS and keloid.

Our previous studies [18, 28] show that IL-10 has potential therapeutic benefits in terms of preventing and reducing HS formation mainly because of IL-10-mediated inhibition of autophagy in HS-derived fibroblasts (HSFs). Therefore, we hypothesized that the abnormal autophagy process in HSFs might underlay the pathological formation of HS. However, the autophagy in dermal fibroblasts, which is one of the most important effector cells responsible for HS formation, is poorly understood. In this study, we first compared the autophagic capacity in HS and normal skin (NS) counterparts and explored the key regulatory molecules of autophagy during the formation of HS. Then, we sought to explore the possible involvement of autophagy and the key regulatory molecules in HS formation. Finally, we utilized gene over-expression, knockdown and silencing technology to verify the key molecular function in HSFs (*in vitro*), and further to validate the protective effects using a rabbit ear scar model (*in vivo*).

## RESULTS

### LC3 was elevated in HS and HSFs

**Figure 1:**
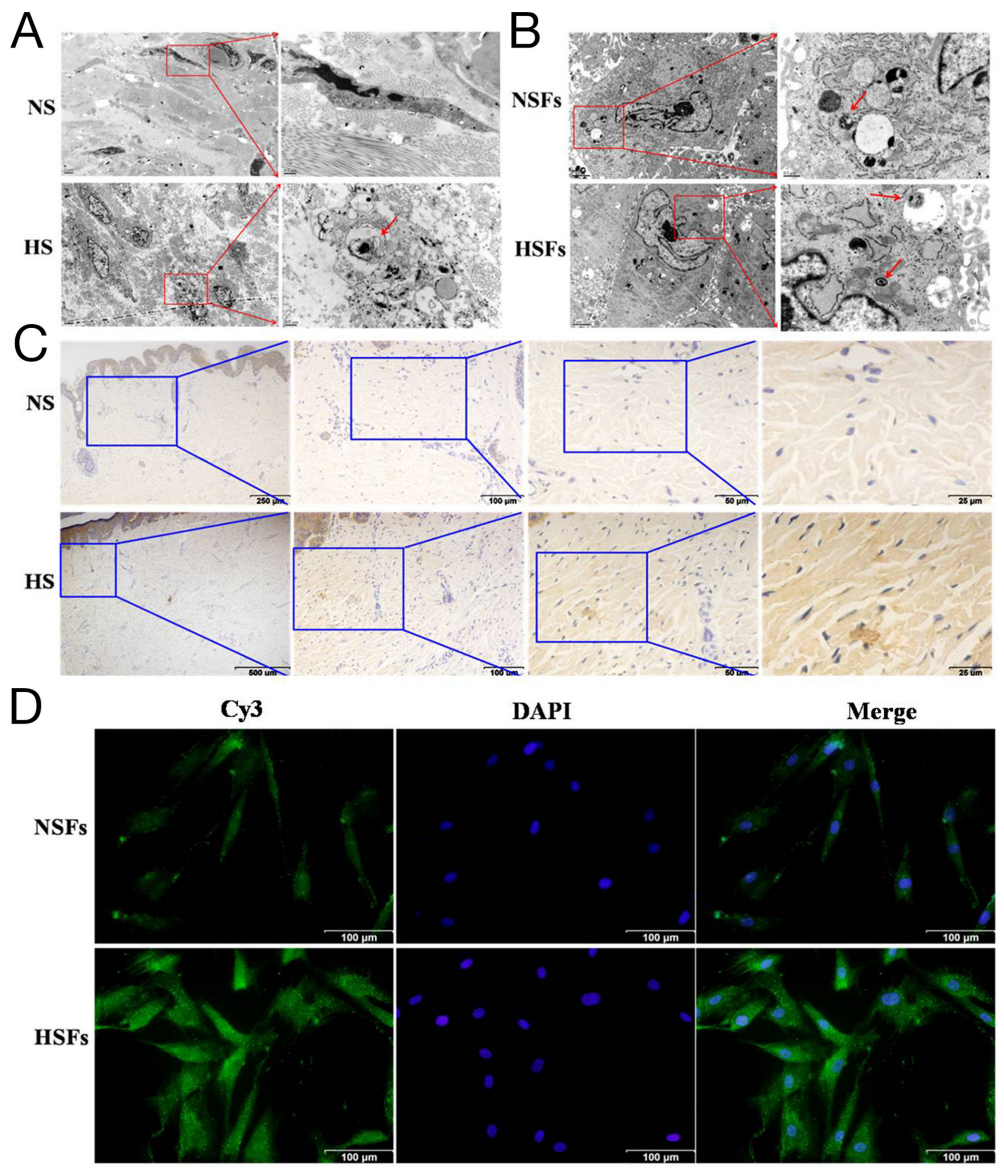
Autophagic capacity was elevated in HS/HSFs **(A)** Tissues were fixed and prepared samples by conventional TEM for observation. There was more autophagosomes in HS (red arrow) than in NS. Scale bars, 2 μm, 0.5 μm. **(B)** Cells were digested, washed, fixed and prepared samples. There were autophagosomes in HSFs similar to in NSFs. Scale bars, 2 μm, 0.5 μm. **(C)** Streptavidin-peroxidase DAB staining showed that LC3 was localized in HS tissue and NS tissue. LC3 was distributed in the cytoplasm, with more intensive staining in HS than in NS tissue. Scale bars, 250 μm, 100 μm, 50 μm, 25 μm. **(D)** NSFs and HSFs were grown on coverslips until they reached 70-80% confluence, fixed in 10% formaldehyde, washed, permeabilized, and blocked. Cells were incubated with an LC3B monoclonal antibody, followed by incubation with a corresponding Cy3-conjugated secondary antibody. The nuclei of the fibroblasts were stained with DAPI. Scale bars, 100 μm.

To verify the differences of autophagic capacity in HS and HSFs, autophagosome was examined using transmission electron microscopy (TEM). Ultra-structure analysis showed that the numbers of autophagosome in the fibroblast of HS was significantly elevated than those in the fibroblast of NS (Figure 1A). But no significantly differences were found in both cultured NSFs and HSFs (Figure 1B). Immunocytochemistry data showed that although LC3 was localized in both NS and HS (Figure 1C), its expression level was significantly higher in HS (Figure 1C). Immunofluorescence results showed that LC3 was localized in cell cytoplasm of NSFs and HSFs (Figure 1D), and significantly higher expressed in HSFs than in NSFs (Figure 1D). These results confirmed that the expression of LC3 in HS/HSFs was higher than that in NS/NSFs.

### LC3 regulated Col 1 and Col 3 expression, and affected α-SMA architecture arrangement in HSFs

**Figure 2:**
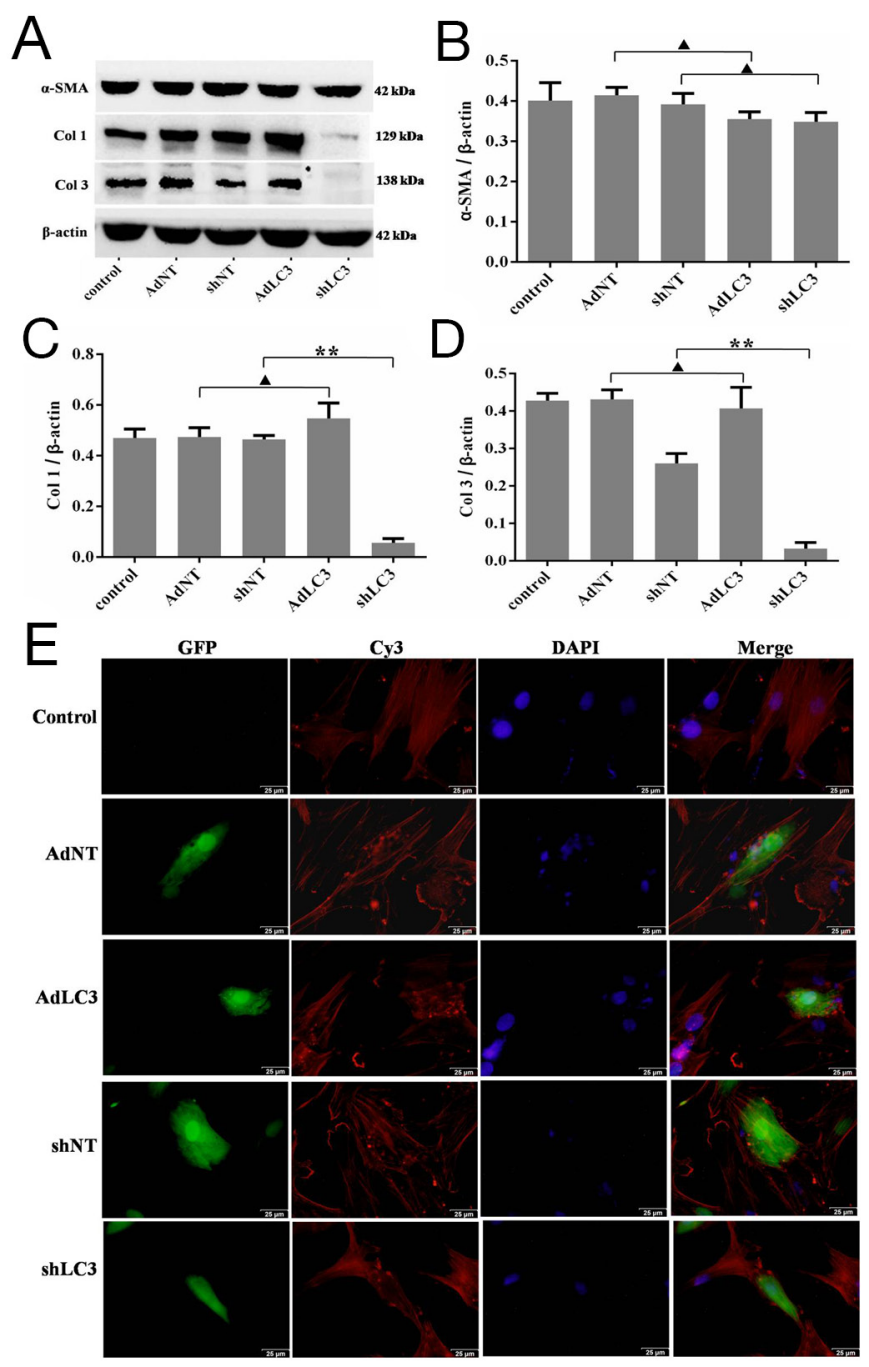
Knockdown of LC3 inhibited the expression of collagen matrix and affects the architecture arrangement of α-SMA in HSFs HSFs, with 50-70% confluent, were infected by AdLC3, shLC3 and their negative control adenvirus vectors, cultured in DMEM medium for 36-48 h, and then protein expression levels were detected by Western blot. **(A, B)** α-SMA protein expresses and changes in the α-SMA/β-actin ratio. **(**A, **C)** Col 1 protein expresses and changes in the Col 1/β-actin ratio. **(**A, **D)** Col 3 protein expresses and changes in the Col 3/β-actin ratio. Data expressed as the mean ± SEM; n = 3, ^▲^p > 0.05, ^*^p < 0.05, ^**^p < 0.01 compared with the negative control. **(E)** The architecture arrangement of α-SMA in infected HSFs (with GFP protein) was analyzed using a specific mAb and a Cy3-conjugated secondary antibody. Fibroblast nuclei were stained with DAPI. Scale bars, 50 μm.

To verify the anti-fibrosis role of LC3 in HSFs, AdLC3 (LC3 over expression), shLC3 (LC3 knock down) and their negative controls (AdNT and shNT) were constituted to infect HSFs in serum-free medium. Western blot analysis showed that knockdown of LC3 (shLC3) blocked the expression of Col 1 (p = 0.0000, Figure 2A and 2C) and Col 3 (p = 0.0002, Figure 2A and 2D), but had no effect on the expression of α-SMA in HSFs (p = 0.1030, Figure 2A and 2B). The up-regulation of LC3 (AdLC3) could suppress neither the expression of Col 1 (p = 0.1492, Figure 2A and 2C) or Col 3 (p = 0.5447, Figure 2A and 2D) nor the expression of α-SMA (p = 0.1760, Figure 2A and 2D) in HSFs.

Alpha-SMA (α-SMA) has been considered a reliable marker for differentiating between fibroblasts and transformed myofibroblasts, as well as the level of tissue fibrosis [29, 30]. To further understand the effect of LC3 on α-SMA, we used immunostaining method to observe the architecture arrangement of α-SMA. Our results showed that in HSFs, α-SMA took the shape of fiber filaments in the control (AdNT and shNT, Figure 2E and 2F) and AdLC3 (Figure 2E) groups, but in shLC3 group, the structure of α-SMA was appeared as a tadpole and rod (Figure 2F). These results implied that knockdown of LC3 (shLC3) not only blocked the expressionof Col 1 and Col 3, but also changed the architecture arrangement of α-SMA (Figure 2F). Knockdown of LC3 (shLC3) might change the function of α-SMA by its architecture arrangement (Figure 2A and 2B), and further affect its function.

### Knockdown of LC3 inhibited the expression of collagen matrix by blocking Bcl-xL in HSFs

**Figure 3:**
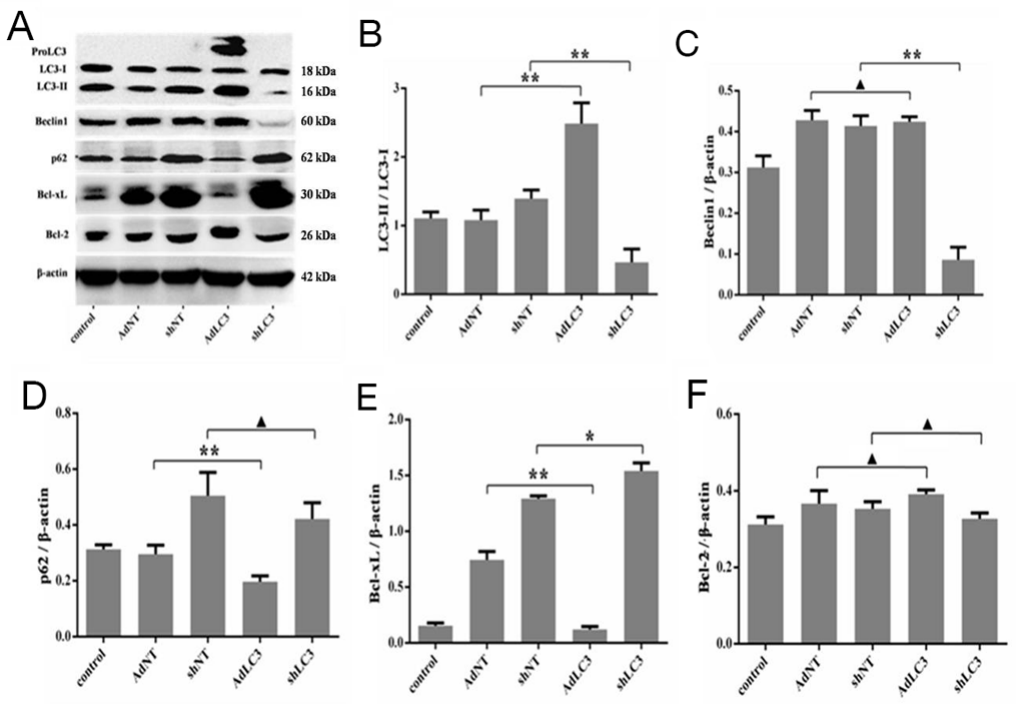
Knockdown of LC3 inhibited both autophagic capacity and the expression of Bcl-xL in HSFs HSFs, with 50-70% confluent, were infected by AdLC3, shLC3 and their negative control adenovirus vectors, cultured in DMEM medium for 36-48 h, and then analyzed with Western blot. **(A, B)** The ratio of LC3-II/LC3-L changes in infected HSFs. **(**A, **C)** auophagy protein Beclin 1 expresses and changes in the Beclin 1/β-actin ratio. **(**A, **D)** Autophagic flux protein p62 expresses and changes in the p62/β-actin ratio.**(**A, **E)** Bcl-xL protein expresses and changes in the Bcl-xL/β-actin ratio.**(**A, **F)** Bcl-2 protein expresses and changes in the Bcl-2/β-actin ratio. Data are representative of three experiments. n = 3, ^▲^p > 0.05, ^*^p < 0.05, ^**^p < 0.01 compared with the negative control group.

It has been reported that Bcl-2 negatively regulates autophagy and autophagic cell death, and Bcl-2 family members may function as oncogenes by blocking apoptosis and autophagy [31-33]. To further understand whether Bcl-2 family through LC3 regulates the expression of collagen matrix in HSFs, we utilized recombinant adenovirus AdLC3 and shLC3 to infect HSFs in serum-depleted medium. As expected, Western blot analysis also showed that AdLC3 upregulated the LC3-II/LC3-I ratio (p = 0.0019, Figure 3A and 3B), downregulated the expression of p62 (p = 0.0107, Figure 3A and 3D) in AdLC3-infected HSFs, but shLC3 had negative or no effect on them (p = 0.0022, p = 0.0001, p = 0.2291, Figure 3A-3D). Moreover, AdLC3 only significantly inhibited the expression of Bcl-xL (p = 0.0002, Figure 3A and 3E), not Bcl-2 (p = 0.0762, Figure 3A and 3F), and shLC3 increased Bcl-xL (p = 0.020, Figure 3A and 3E), not Bcl-2 (p = 0.1262, Figure 3A and 3F). These results implied that shLC3 blocked the expressionof Col 1 and Col 3 in HSFs by Bcl-xL, but not by Bcl-2.

In view of the above results, we also detected the expression level of Bcl-xL in HS/NS and HSFs/NSFs. Immunocytochemistry results showed that Bcl-xL localized in both HS/NS and HSFs/NSFs, with the expression level was significantly higher in HS/HSFs than in NS/NSFs, respectively (Supplementary Figure 1A and 1B).

### Silencing for Bcl-xL inhibited the expression of collagen matrix

To further test shLC3 blocked collagen matrix expression by Bcl-xL, sibcl-xL and sibcl-2 were applied to knockdown Bcl-xL and Bcl-2 in HSFs, followed by observing the effect of them on collagen matrix. We firstly validated the effects of sibcl-xL and sibcl-2 on their protein levels in HSFs, and Western blotting showed that sibcl-xL and sibcl-2 inhibited the target protein expression (Supplementary Figure 2A, 2B and 2C).

**Figure 4:**
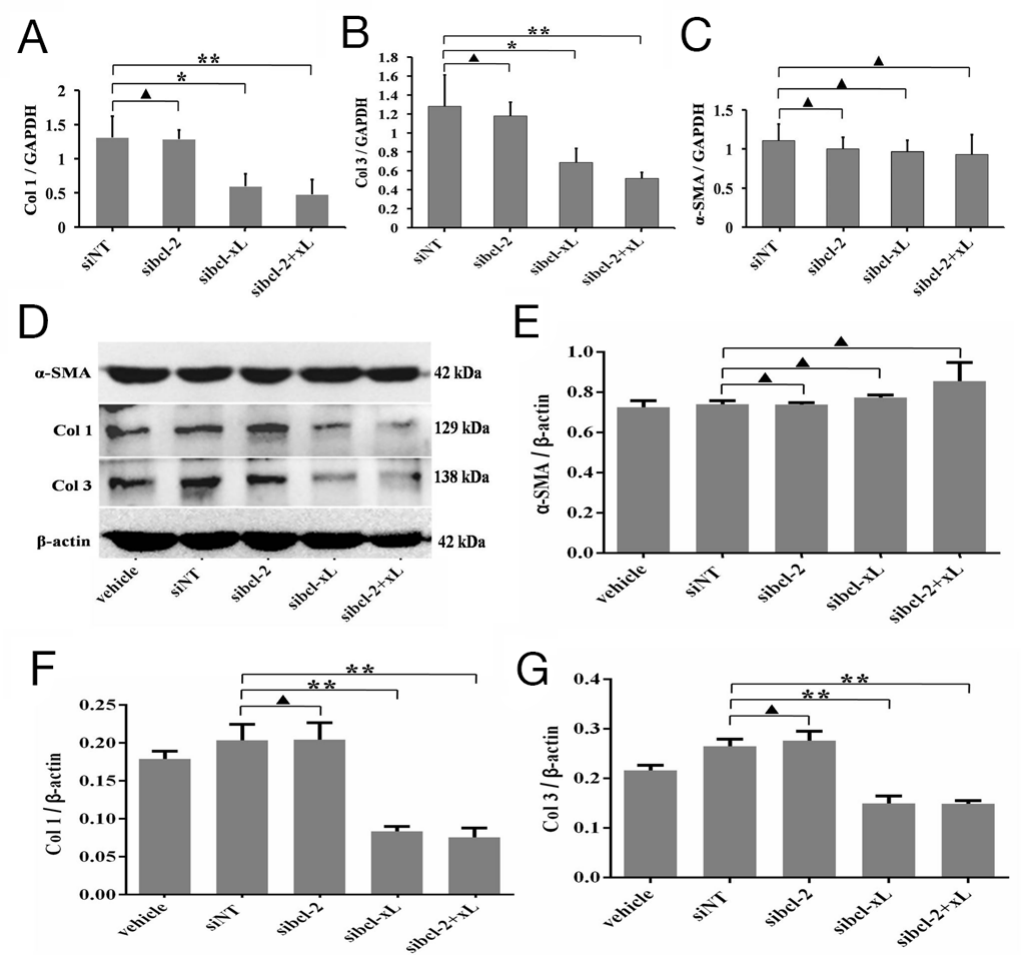
Silencing for Bcl-xL inhibited the expression of collagen matrix in HSFs HSFs, with 70-80% confluent, were transfected by siRNAs and their negative control, cultured in DMEM medium for 24 h and 48 h, then qRT-PCR and Western blot analyses. **(A, B, C)** The mRNA level of Col 1, Col 3 and α-SMA mRNA was analyzed by qRT-PCR and normalized against GAPDH. **(D, E)** α-SMA protein expresses and changes in the α-SMA/β-actin ratio. **(**D, **F)** Col 1 protein expresses and changes in the Col 1/β-actin ratio. **(**D, **G)** Col 3 protein expresses and changes in the Col 3/β-actin ratio. Data are representative of three experiments. n = 3, ^▲^p > 0.05, ^*^p < 0.05, ^**^p < 0.01 compared with the negative control group.

Then, quantitative PCR (qRT-PCR) analysis showed that sibcl-xL could suppress the transcription levels of Col 1 (p = 0.0126, Figure 4A) and Col 3 (p = 0.0157, Figure 4B) in HSFs, but sibcl-2 could not (p = 0.1941 and p = 0.1213, Figure 4A and 4B). Neither sibcl-xL nor sibcl-2 changed the transcription level of α-SMA (p = 0.1272 and p = 0.1535, Figure 4C). Western blot analysis showed that sibcl-xL, but not sibcl-2 (p = 0.9604 and p = 0.4407, Figure 4D, 4F and 4G), remarkably suppressed the expression of Col 1 (p = 0.0007, Figure 4D and 4F) and Col 3 (p = 0.0007, Figure 4D and 4G) in HSFs. Neither sibcl-xL nor sibcl-2 changed the expression of α-SMA (p = 0.0597 and p = 0.8511, Figure 4D and 4E). All these results implied that Bcl-xL was a key signaling molecule involved in collagen metabolism in HSFs.

### Silencing for Bcl-xL suppressed autophagic capacity in HSFs, and promoted apoptosis of HSFs

**Figure 5:**
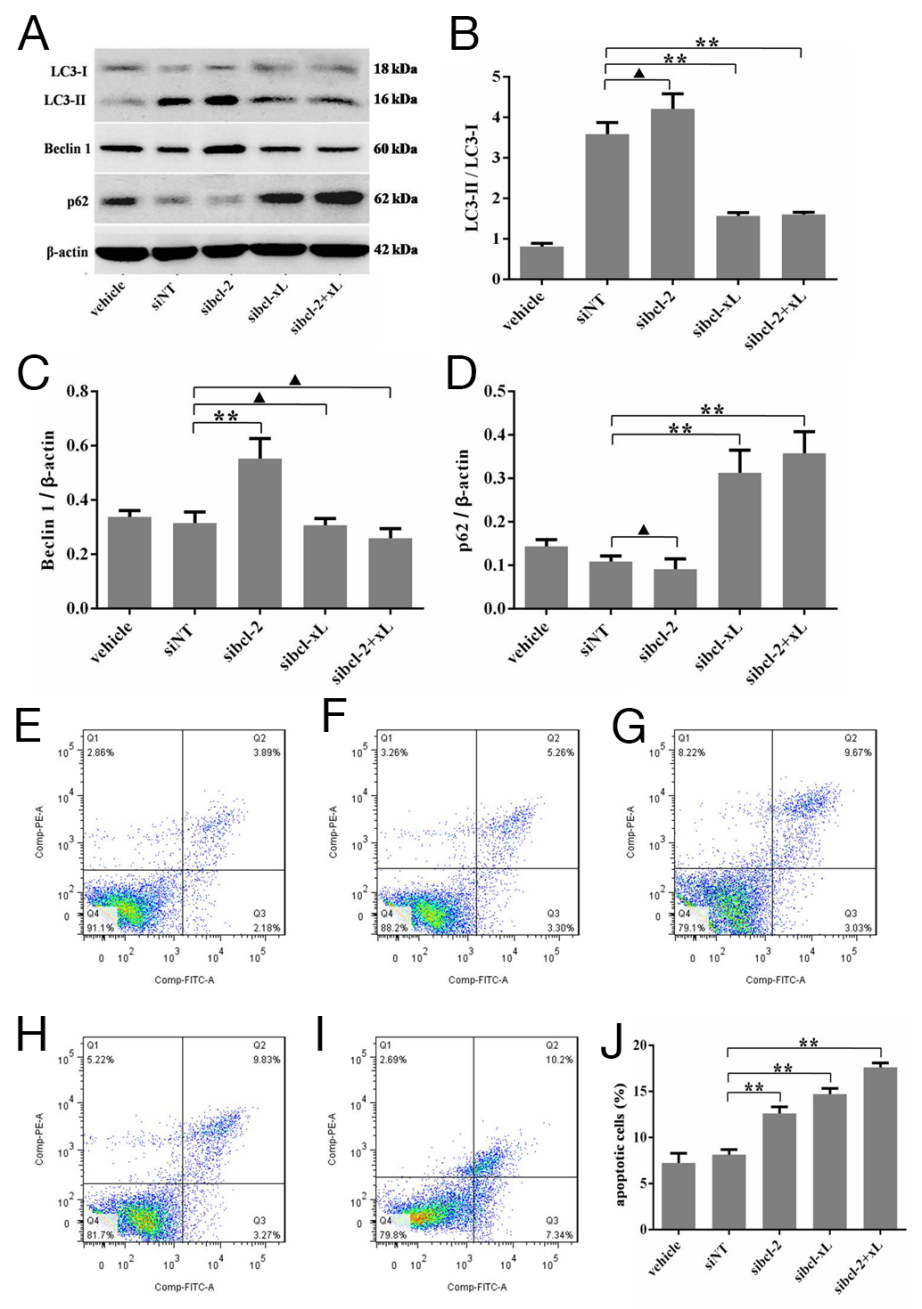
Silencing for Bcl-xL inhibited the autophagic capacity in HSFs, and promoted the apoptosis of HSFs HSFs, with 70-80% confluent, were transfected by siRNAs and their negative control, cultured in DMEM medium for 48 h, then Western blot analyses. **(A, B)** The ratio of LC3-II/LC3-L changes in transfected HSFs. **(**A, **C)** Autophagy protein Beclin 1 expresses and changes in the Beclin 1/β-actin ratio. **(**A, **D)** p62 protein expresses and changes in the p62/β-actin ratio. **(E-J)** HSFs, with 70-80% confluent, were transfected by siRNAs and their negative control, cultured in DMEM medium for 48 h. Apoptosis was measured by flow cytometric analysis of Annexin V and propidium iodide staining. Blank cells were used as control. Data are representative of three experiments. n = 3, ^▲^p > 0.05, ^*^p < 0.05, ^**^p < 0.01 compared with the negative control group.

It is well known that the LC3-II/LC3-I ratio is indicative of autophagic capacity. So, the changes of the LC3-II/LC3-I ratio in HSFs after Bcl-xL and Bcl-2 silencing were checked. Convincingly, Western blotting results showed that sibcl-2 could, at least to some extent, increase the LC3-II/LC3-I ratio (p = 0.0808, Figure 5A and 5B), sibcl-xL could apparently decrease the LC3-II/LC3-I ratio (p = 0.0003, Figure 5A and 5B), sibcl-xL+sibcl-2 (sibcl-2+xL) could also decrease the LC3-II/LC3-I ratio (p = 0.0003, Figure 5A and 5B). As for Beclin 1, it could be downregulated only in sibcl-2 group (p = 0.0080, Figure 5A and 5C). Moreover, the expression of p62 (Figure 5A and 5D) also supported the above results. Taken together, these results confirmed that Bcl-xL was a key signaling molecule involved in autophagy in HSFs.

In addition, a flow-based Annexin V assay was conducted the effect of sibcl-xL-mediated LC3 silencing on HSFs apoptosis. As shown in Figure 5E-5J, the sibcl-xL promoted apoptosis levels in HSFs subsequent to 48 h transfection, and the apoptosis rates of Lipofectamine RNAiMAX Reagent alone, negative sequence transfection, sibcl-xL, sibcl-2 and sibcl-2+xL groups were 7.22 ± 0.62% (Figure 5E), 8.12 ± 0.32% (Figure 5F), 12.60 ± 0.42% (Figure 5G), 14.71 ± 0.36% (Figure 5H) and 17.59 ± 0.29% (Figure 5I) respectively. Among them, the apoptosis rates of HSFs transfected with sibcl-xL, or sibcl-2, either alone or in combination, were significantly elevated when compared with those of the control groups (p = 0.0002 and p = 0.0011, Figure 5J). It seemed that sibcl-xL played a stronger role in promoting apoptosis compared with sibcl-2. These results further confirmed that Bcl-xL was also a key signaling molecule involved in apoptosis of HSFs.

### Knockdown of autophagy protein LC3 (shLC3) via blocking the expression of Bcl-xL (sibcl-xL) inhibited scar formation on the rabbit ear

**Figure 6:**
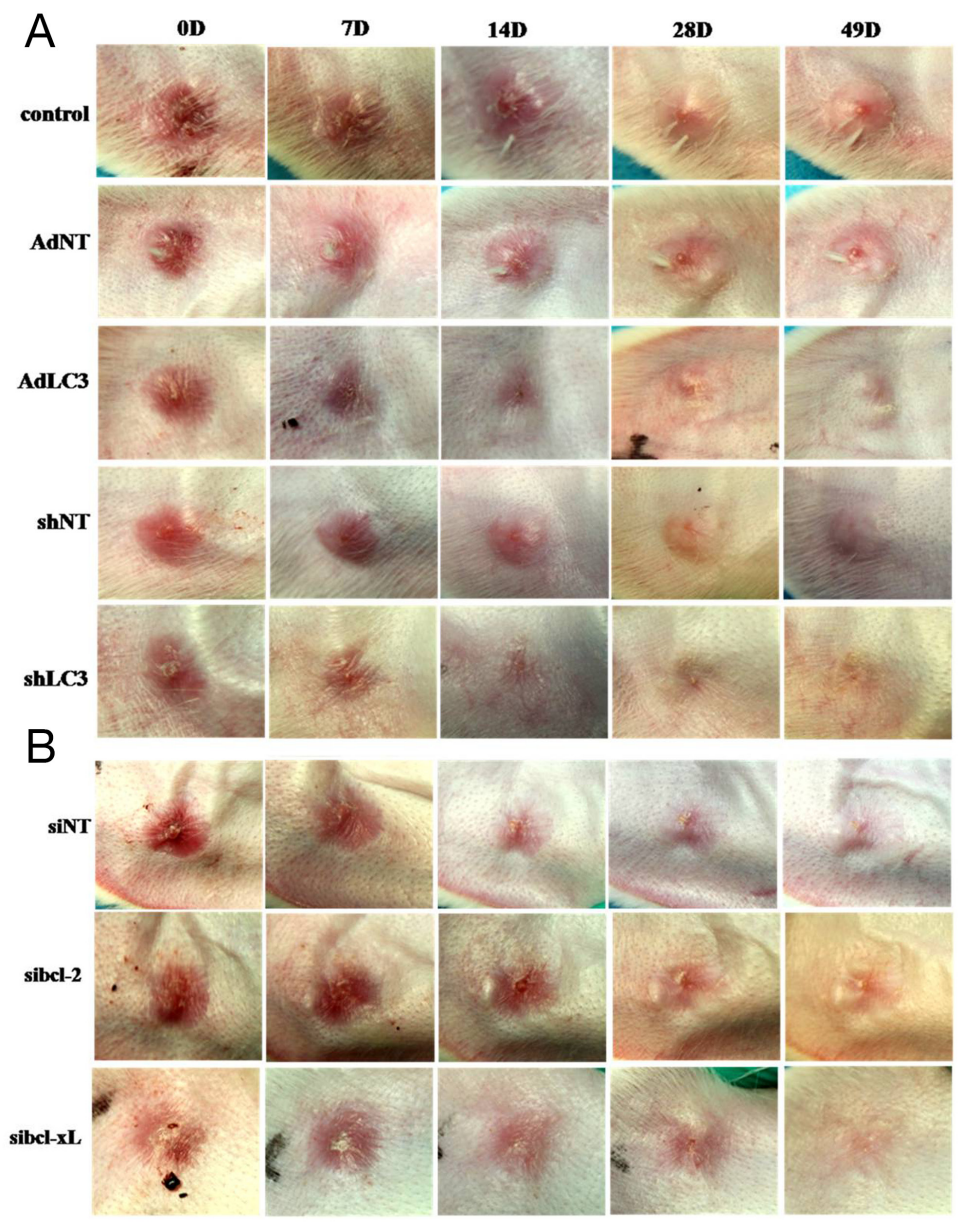
Knockdown of LC3 or Silencing for Bcl-xL improved the appearances of rabbit scar model The rabbits were anaesthetized and created four full-thickness wounds which were then covered with sterile gauze. On postoperative day 28 and afterwards, scars were randomly placed into eight groups: one PBS group, three control groups (AdNT, shNT and siNT) and four treatment groups (AdLC3, shLC3, sibcl-2 and sibcl-xL). They were applied to the scars two times in a week. After treatment 49 days, **(A)** the pictures of scar appearance were shown that the scar appearance in shLC3 were smaller and flatter than in AdLC3 and its control. **(B)** The sibcl-xL group was also smaller and flatter than in sibcl-2 and its control (n = 6).

**Figure 7:**
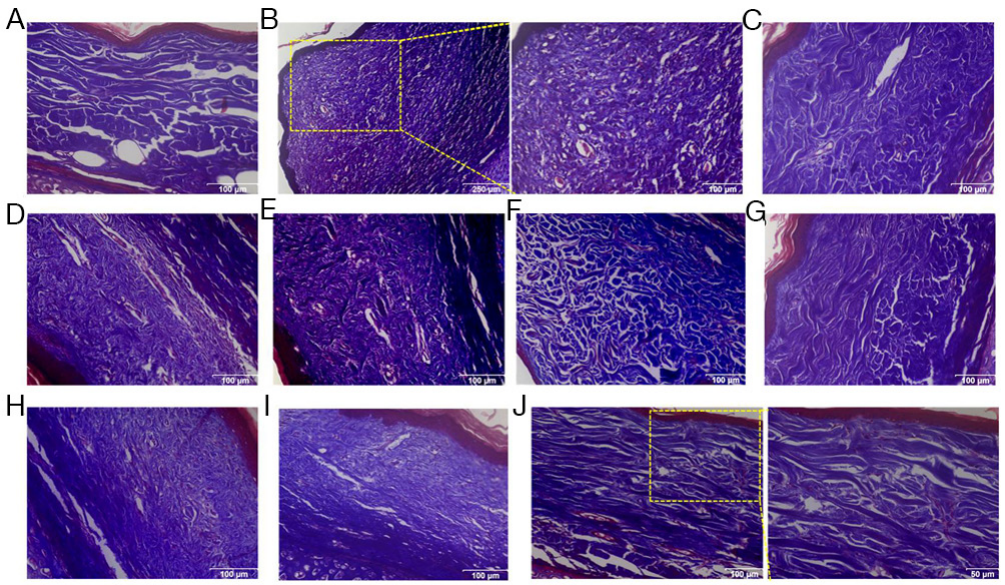
Knockdown of LC3 or Silencing for Bcl-xL reduced the deposition and improved the arrangement of collagen fibers in rabbit scar model Masson’s trichrome staining findings for collagen fibers. **(A)** Rabbit ear normal skin with regularly arranged, small collagen fibers. **(B)** Rabbit ear scar model tissue with thicker, denser and disorganized collage fibers. **(C-F, H, I)** In PBS, AdNT, AdLC3, shNT, siNT and sibcl-2 groups, the amount of collagen fibers is far more than that in the normal group, and collage fibers are thicker, denser and disorganized. **(G, J)** After treatment with shLC3 and sibcl-xL for 49 days, the collagen fibers were arranged more regularly and sparser than those in the model, PBS and corresponding control groups. n = 6 scars in each group. Scale bars, a, c-i, 100 μm; b, 250 μm, 100 μm; j, 50 μm.

Our *in vitro* study has demonstrated that LC3 regulated HSFs fibrosis by controlling the expression of Bcl-xL. To further confirm the effects of LC3 and its regulating molecule Bcl-xL on the continuous process of wound healing and scar formation, cutaneous excision wound models were established in Netherland rabbit, which were treated with i.d. injections of AdLC3, shLC3, sibcl-xL and sibcl-2. Convincingly, the scar appearance after treatment in shLC3 (Figure 6A) and sibcl-xL (Figure 6B) groups were smaller and flatter than those in AdLC3 (Figure 6A), sibcl-2 (Figure 6B) groups and their controls (Figure 6A and 6B). And more, *Masson* trichrome *staining* showed that collagen fibers in rabbit ear scar model tissue (Figure 7B) were more disordered structure and denser compared to normal rabbit ear tissue controls (Figure 7A), and that shLC3 (Figure 7G) and sibcl-xL (Figure 7J) treatment resulted in more neatly arranged and thinner scar appearance compared with the control groups (PBS and vehicle, Figure 7C, 7D, 7F and 7H). In contrast, the AdLC3 led to more disordered structure and denser collagen fibers (Figure 7E) compared with shLC3 group (Figure 7G), and sibcl-2 (Figure 7I) compared with sibcl-xL group (Figure 7J), and the latter was similar to the normal tissue (Figure 7A). These results confirmed that knockdown of LC3 (shLC3) via blocking the expression of Bcl-xL (sibcl-xL), which was a key molecule involved in collagen metabolism, inhibited scar formation on the rabbit ear (*in vivo*).

## DISCUSSION

HS is a pathologically significant skin fibrotic disease and the major characteristics of HS are the over-proliferation and activation of dermal fibroblasts, as well as the metabolic disorder of collagen-based ECM proteins [18-22, 34-36]. HS formation usually results from disturbance of the tightly controlled tissue repair mechanism due to traumatic skin injury. HS is not only aesthetically displeasing, but also obstructs normal muscle function, thereby contributing to psychological and physical suffering [15-18]. Statistically, the incidence of HS ranges from 40-70% following surgery, and up to 91% following burn injury [36]. Currently, no effective therapeutically relevant targets have been reported for HS treatment largely due to the undefined molecular mechanism. During the normal wound healing process, fibroblasts around the wound are activated and transdifferentiated into myofibroblasts. Then they migrate into the wound area, and facilitate wound healing by increasing the synthesis and secretion of collagen. After wound healing, myofibroblasts are removed by apoptosis [19, 30, 37]. However, under pathological conditions, such as prolonged inflammation and infection, myofibroblasts persist in the wound, result in the excessive deposition of ECM proteins and HS formation [19, 30, 37]. Thus, the inhibition of collagen synthesis/secretion and promoted apoptosis of myofibroblasts are rather important for the treatment of HS.

Dermal fibroblasts, one of the most important effector cells responsible for HS formation, are usually in a quiescent status in NS while become activated after skin injury [38]. Aberrant proliferation of fibroblasts and excessive deposition of ECM often result in scarring [35]. Compared to the scarless healing, persistent activation of fibroblasts and increasing deposition of ECM were usually observed in pathological scars [34, 36]. As collagen is one of the key components in ECM, the continuous expression of Col 1 and Col 3, and the imbalance of Col 1/Col3 ratio are important histological features to distinguish HS from NS [35, 39, 40]. In addition, activated fibroblasts overexpress α-SMA, which is a well-known marker for myofibroblasts and promotes scar contraction [41]. Therefore, the regulation of fibroblast proliferation/activation and ECM protein synthesis are the main focus to study the prevention and treatment of HS.

Currently, there is no effective therapy for HS, largely because the mechanisms underlying HS development are poorly understood [35, 42]. Recently, more and more studies [3-10] show that dysregulation of autophagy associates with the pathogeneses of many diseases in mammals because autophagy is a lysosomal degradation pathway essential for cellular survival, differentiation, development, and homeostasis [5-8], and autophagy principally serves an adaptive role to protect organisms from pathogen infection, aging, neurodegeneration, and cancer during disease pathogenesis [9, 10, 23-26]. LC3, an indicator of autophagy induction in mammals, is an autophagosomal ortholog of yeast Atg8 and it is derived from a posttranslational cleavage of carboxyl terminus amino acid residue of its precursor proLC3. This modification process results in the exposure of a carboxyl terminal glycine in LC3 and, therefore, turns it into a soluble form LC3-I [43, 44]. Upon starvation, LC3-I is further modified to LC3-II, which has greater electrophoresis mobility than LC3-I. Therefore,Conversion of LC3-I to LC3-II correlates with the formation of autophagosomes [13, 14, 45]. And the changes in the LC3-II/LC3-I ratio are indicative of autophagic capacity [13, 14, 45]. Our previous studies [27, 28] show that autophagy plays an important role in HS formation. In this study, our results showed that LC3 positive staining and autophagosomes in HS was more intensive relative to those in NS group (Figure 1, 2). Knockdown of LC3 (shLC3) inhibited the expressionof Col 1 and Col 3 (Figure 3), and changed the architecture arrangement of α-SMA in HSFs. But AdLC3 could not affect the expressionof Col 1, Col 3 or α-SMA in HSFs (Figure 3). Interestingly, our results implied that shLC3 blocked autophagy through Bcl-xL, but not Bcl-2 (Figure 4), and showed that Bcl-xL was a key signaling molecule involved in autophagy in HSFs.

Bcl-xL, an antiapoptotic member of the Bcl-2 family, is a transmembrane molecule in the mitochondria, and acts as a pro-survival protein by preventing the release of mitochondrial contents [46-48]. *Bcl*-2, an inner mitochondrial membrane protein, is unique among proto-oncogenes, being localized to mitochondria and interfering with programmed cell death independent of promoting cell division [46-48]. Many studies [49, 50] have reported that Bcl-2 is the prototypical inhibitor of apoptosis (type I programmed cell death) and is upregulated in 75% of pancreatic cancers, and that the high levels of Bcl-2 in pancreatic cancer confer a potent antiapoptotic signal as well as confer chemoresistance, and that Bcl-2 also regulates autophagy in pancreatic cancer [51]. To validate whether LC3 regulate the expression of collagen matrix by Bcl-2 and Bcl-xL, we utilized siRNA to silence Bcl-2 and Bcl-xL to check the changes of fibrotic factors and autophagy in HSFs. The results implied that sibcl-xL, but not sibcl-2, blocked the expressionof fibrotic factors (such as Col 1 and Col 3) and changed the architecture arrangement of α-SMA (Figure 4 and 2), and inhibited the autophagy (Figure 5) in HSFs. Moreover, sibcl-xL could increase the apoptotic function of HSFs (Figure 5). Based on these findings, we proposed LC3 and Bcl-xL being attractive anti-HS candidate targets. More persuasively, Rabbit ear scar models injected either shLC3 or sibcl-xL resulted in smaller and flatter scar appearance (Figure 6), and in more neatly arranged and thinner morphology compared with the control groups (Figure 7). These findings thus suggested Bcl-xL as a potential molecular target, and disrupting autophagy by manipulating Bcl-xL inactivation status (not Bcl-2), might be an effective method to treat HS and related fibrotic diseases.

It is worth noticing that although immunofluorescence analysis showed shLC3 could not significantly inhibit the expression of α-SMA, it mainly changed the architecture arrangement and function of α-SMA. Because in AdLC3-infected HSFs, the structure of α-SMA took the shape of clavite (Figure 2E), but not as the structure of fibers as in untreated HSFs (Figure 2E). Knockdown of LC3 (shLC3) changed the function of α-SMA because shLC3 did not affect the expression of α-SMA in HSFs, but shLC3 resulted in more neatly arrangement (Figure 7). The results implied that both shLC3 and sibcl-xL blocked the expressionof Col 1 and Col 3. But they were different in changing the function of α-SMA. That is to say shLC3 could not only decrease the expression of Col 1 and Col 3, but also changed the function of α-SMA, repress the transformation of fibroblasts to myofibroblasts, so as to protect against HS fibrosis effect.

Since HS is a pathologically fibro-proliferative disorder of dermal wound healing characterized by excessive ECM accumulation, the exaggerated deposition and attenuated degradation of Col 1 and Col 3 are the major cause during HS formation [18-22, 34-36]. Based on above findings, we were eager to reveal if the abundant collagen deposition could be effectively attenuated by manipulating LC3 and Bcl-xL. Combining gene over-expression, knockdown and silencing, our results revealed that only suppression of LC3 and Bcl-xL could obviously downregulate the expression levels of both Col 1 and Col 3, reduced ECM accumulation (Figure 2 and 4), and ameliorated collagen arrangement (Figure 7). Suggesting the synergistic action of LC3 and Bcl-xL on HS pathogenesis, and moreover, silencing for Bcl-xL was found to increase the apoptosis of HSFs (Figure 5), and reduce the formation of HS after wound closing in a rabbit ear scar model (Figure 6 and 7).

In conclusion, we identified a novel mechanism by which autophagy protein LC3 regulated the fibrosis of HS by controlling the expression of Bcl-xL *in vitro* (in HSFs) and *in vivo* (in rabbit ear scar model). The aberration of LC3 protein processing compromised autophagy in HS might associate with the pathogenesis of HS in wound healing because ① It was by Bcl-xL, but not Bcl-2, that LC3 regulated the expression of Col 1 and Col 3 in HSFs to block the secretion and accumulation of ECM in order to inhibit HS formation and dermal fibrosis. ② sibcl-xL significantly increased the apoptosis of HSFs comparing to sibcl-2, better balance the excessive proliferation of the fibroblasts in dermal skin. ③ Although LC3 via Bcl-xL could not change the expression of α-SMA, but shLC3 significantly improved the architecture arrangement and function of α-SMA, which might change the function of myofibroblasts, so as to reduce ECM secretion, the scar contraction and skin fibrosis. As for the further mechanism of LC3 via Bcl-xL improved the arrangement and function of α-SMA, of cause, this need to be further studied. In a word, Bcl-xL could serve as a key molecular target, and might be a novel strategy for HS therapy. Meanwhile, our findings might shed more light on the molecular mechanism underlying HS formation and highlight the therapeutic potential of HS.

## MATERIALS AND METHODS

### Collection and processing of HS tissues

HS and normal dermal skin (NS) tissues [18, 28] were collected from patients who had undergone surgical excision at Xijing Hospital (Xi’an). Written consents were obtained from all participants before surgery. All protocols used in the study were approved by the Ethics Committee of Xijing Hospital, affiliated to the Fourth Military Medical University of China. Each collected skin tissue sample was split into two portions: one portion was preserved in 10% buffered formalin solution for immunostaining and the remaining portion was to isolate fibroblasts for culture.

### Immunostaining

Immunostaining wasperformed as previously reported [18, 27, 28]. In brief, the skin tissues fixed in 10% buffered formalin were embedded in paraffin blocks and cut into 4 μm-thick tissue sections. The processed tissue sections were then dewaxed and treated with 3% hydrogen peroxide for 15 min, followed by blocking with goat serum for 30 min, incubation at 4°C overnight with a primary monoclonal antibody (mAb) against LC3B (1:100, Cell Signaling) and Bcl-xL (1:100, Cell Signaling), and immunostained with a SP-9000 Histostain™ Kit (ZSGB; SP-9000D), according to the manufacturer’s instructions. Briefly, tissue sections/cells on slide were incubated with a biotinylated secondary antibody, treated with streptavidin-biotin-horseradish peroxidase for signal amplification, and then stained with diaminobenzidine (DAB). Finally, the tissue sections/cells on slide were counterstained with hematoxylin. Isotype-matched IgG was used as a negative control for each immunostaining procedure.

Immunofluorescence analysis was performed as previously reported [18]. In brief, cells were grown on coverslips for 24-36 h until 70-80% confluent, fixed in 4% formaldehyde for 30 min, washed with phosphate buffered saline (PBS), permeabilized with 0.1% Triton-X100 for 10 min at room temperature, blocked with 1% bovine serum albumin (BSA), hybridized with a rabbit mAb specific for LC3B (1:100, Cell Signaling) at room temperature for 1 h, and then incubated with a Cy3-conjugated goat anti-mouse secondary antibody (1:100 dilution; Cwbio) at 37°C for 1 h. Finally, the samples were stained with 4’,6’-diamidino-2-phenylindole (DAPI, Sigma).

### Transmission electron microscopy (TEM)

Ultrathin sections of HSFs were processed in conventional methods [28]. The samples were fixed with 2.5% glutaraldehyde in PBS buffer, included in agar, rinsed in Sorensen’s buffer, postfixed in 1% osmium tetroxide in Sorensen’s buffer, dehydrated in ethanol, and embedded in EPON resin. Ultrathin sections were cut and stained with uranyl acetate and lead citrate, and examined using a JEM-123 transmission electron microscope (JEOL) at 80 kV.

### Cell culture and treatment

Cell culture was performed as previously described [18, 28]. Briefly, fibroblasts were extracted from minced HS tissues by incubation in a solution of collagenase type I (0.1 mg/mL; Sigma) at 37°C for 2.5 h. Extracted HSFs were collected and cultured at 37°C (in a 5% CO_2_-humidified incubator) in Dulbecco’s Modified Eagle’s Medium (DMEM, Gibco) supplemented with 10% fetal calf serum (FCS, Gibco), 100 U/mL penicillin, and 100 U/mL streptomycin (Hyclone). All experiments were performed with cells at passage 3-5. Biochemical analysis was conducted on HSFs at 70-80% confluence after incubation for 12-16 h in serum-free medium.

### Infection of overexpression (Ad-) and knockdown (sh-) adenovirus vectors, and the transfection of silencing for RNA (siRNA)

The recombinant adenovirus vector for LC3B (AdLC3), knockdown LC3B (shLC3) and the control adenvirus vector containing non-targeting (AdNT) shRNA (shNT) were purchased from Genomeditech Company (Genomeditech, Shanghai, China). All vectors were labeled with GFP, which served as a detecting marker. HSFs, grown to 50-70% confluence, were incubated for 12 h in serum-free medium and infected with vector for 48 h to generate stable cells. As for siRNA (GenePharma, Shanghai, China) transfection, HSFs were seeded in complete media in 6-well dishes the day before the experiment. For all assays, the concentration of siRNAs during transfection was 10 nM. All transfections were performed in a mixture of Opti-MEM and complete media without antibiotics, as described previously [52, 53]. The siRNA duplexes for Bcl-xL, Bcl-2 or scramble control sequences were listed in Table 1. The transfection incubation time for siRNA/Lipofectamine RNAiMAX reagent complexes (Lifetechnologies) was 24 h. The total incubation time before cell lysis and protein isolation was 48 h. The efficiency of transfection was measured by Western blot analysis.

**Table 1:** Sequences of siRNA

Type of gene	Forward (5’→3’)	Reverse (5’→3’)
Bcl-2	CCCUGUGGAUGACUGAGUATT	UACUCAGUCAUCCACAGGGTT
Bcl-xL	AUUGGUGAGUCGGAAUGGCATT	UGCGAUCCGACUCACCAAUTT
Nagative control	UUCUCCGAACGUGUCACGUTT	ACGUGACACGUUCGGAGAATT

### qRT-PCR

qRT-PCR was performed as previously reported [18, 28, 52, 53]. In brief, total RNAs were extracted from cultured cells using an RNA isolation kit (Takara). The purity of the RNA was calculated as follows: A260/A280 (1.9-2.0). The primer pairs (human) used for gene amplification from the cDNA template were listed in Table 2. The relative expression of the target gene transcripts was expressed as the mean abundance from three independent reactions. Expression of target gene was normalized against that of GAPDH.

**Table 2:** Primers of qRT-PCR

Type of gene	Forward (5’→3’)	Reverse (5’→3’)
Col 1	GAGGGCAACAGCAGGTTCACTTA	TCAGCACCA CCGATGTCCA
Col 3	CCACGGAAACACTGGTGGAC	GCCAGCTGCACATCAAGGAC
α-SMA	GACAATGGCTCTGGGCTCTGTAA	TGTGCTTCGTCACCCACGTA
GAPDH	GCACCGTCAAGCTGAGAAC	TGGTGAAGACGCCAGTGGA

### Western blot analysis

Cultured HSFs were harvested, washed in PBS, and resuspended in RIPA cell lysis solution (Beyotime) supplemented with 200 μg/mL phenylmethylsulfonyl fluoride (PMSF, Boster), phosphatase inhibitor cocktail (Sigma), and protease inhibitor cocktail (Sigma). The protein concentration of the cell lysates was determined using the BCA assay (Pierce).

Western blotting was performed as previously described [18, 28, 52, 53]. Briefly, cell lysates containing equal amounts of protein were separated in 8% (for Col 1 and Col 3) or 14% (for LC3) SDS-PAGE gels and transferred to polyvinylidene fluoride (PVDF, Millipore) membranes at 100 V for 30 min (for LC3, Bcl-2 and Bcl-xL) or 100 min (for Col 1, Col 3, Beclin1). Membranes were then blocked with 5% non-fat milk in TBST (tris buffered saline/0.5% Tween-20) at room temperature for 3 h, followed by incubation at 4°C overnight with rabbit/mouse mAbs specific for Bcl-2 (Cell Signaling, 15071), Bcl-xL (Cell Signaling), Col 1 (Abcam), Col 3 (Abcam), alpha-actin (Epitomics), LC3B (Cell Signaling). Finally, the membranes were washed and incubated with HRP-conjugated secondary antibodies (1:3,000 dilution, Bioss). The immunoreactive protein bands were detected using ECL reagents (Millipore). The signal intensity of each protein was quantified by scanning the membrane with an image analyzer (Alpha Innotech). The membrane was then stripped of antibodies and re-probed with a rabbit mAb against β-actin (1:2,000 dilution, Cell Signaling) as an internal loading control.

### Rabbit ear scar model and treatment

We used a previously described rabbit ear scar model in this study [53, 54]. New Zealand white rabbits of male weighing 2.0-2.5 kg were purchased from the Experimental Animal Center of the Fourth Military Medical University and were maintained in separate cages. The animal experiments were approved by the Experimental Animal Committee of the Fourth Military Medical University. The animals were anaesthetized by intravenous administration of sodium pentobarbital (30 mg/kg). In a sterile environment, four full-thickness wounds down to the cartilage on each ear were created. Four round wounds, 10 mm in diameter, were randomly created on the ear. Each rabbit thus had 8 wounds. For each wound, the epidermis, dermis and perichondrium were completely removed. The wounds were then covered with sterile gauze for 1 day. The rabbits were returned to their cages after they recovered from anesthesia. On postoperative day 28 and afterwards, scars were randomly placed into eight groups (6 scars to each group): one PBS group, two control groups (AdNT and shNT), siRNA control (siControl/siNT) and four treatment groups (AdLC3, shLC3, sibcl-2 and sibcl-xL). They were applied to the scars two times in a week.

### Flow cytometry

FITC Annexin V Apoptosis Detection Kit (BD Pharmingen™, Cat: 556547) was used to measure cell apoptosis after transfection [29, 55, 56]. Briefly, the cells were treated with medium alone or in the presence of siRNA specific for Bcl-xL or with negative control siRNA or with Lipofectamine RNAiMAX Reagent (Lifetechnologies) only for 48 h. The cells were washed in PBS, resuspended in 100 μl of binding buffer and stained with 5 μl FITC-Annexin V, and 5 μl propidium iodide (PI) for 15 min in the dark, then added 300 μl of binding buffer, according to the manufacturer’s instructions. Analyses were performed with flow cytometry (BD FACSAria™ III system; BD Pharmingen). The cells in the FITC-positive fraction were regarded as apoptotic cells.

### Statistical analysis

Quantitative data are expressed as the mean ± standard error of the mean (SEM). Student’s t-test was used to compare data between two groups and analysis of variance (ANOVA) was used for multiple-group comparisons. A value of p < 0.05 was considered statistically significant.

## SUPPLEMENTARY MATERIALS FIGURES



## Abbreviations

ANOVA: analysis of variance
BCA: bicinchoninic acid
BSA: bovine serum albumin
Col 1: type I collagen
Col 3: type III collagen
DAB: diaminobenzidine
DAPI: 4’,6’-diamidino-2-phenylindole
DMEM: Dulbecco’s modified Eagle’s medium
ECL: enhanced chemiluminescence
ECM: extracellular matrix
FCS: fetal calf serum
GAPDH: glyceraldehyde-3-phosphate dehydrogenase
GFP: green fluorescent protein
HRP: horseradish peroxidase
HS: hypertrophic scar
HSFs: hypertrophic scar derived fibroblasts
IgG: immunoglobulin G
mAb: monoclonal antibody
LC3: microtubule-associated protein 1 light chain 3
NS: normal skin
NSFs: normal skin derived fibroblasts
PBS: phosphate-buffered saline
PMSF: phenylmethylsulfonyl fluoride
PVDF: polyvinylidene fluoride membrance
qRT-PCR: quantitative real-time polymerase chain reaction
SDS-PAGE: sodium dodecyl sulfate-polyacrylamide gel electrophoresis
SEM: standard error of the mean
TBST: tris buffered saline/0.5% tween-20
TEM: transmission electron microscopy
