# Supplementary material for: Autophagy protein LC3 regulates the fibrosis of hypertrophic scar by controlling Bcl-xL in dermal fibroblasts
Source: Oncotarget. 2017 Sep 8;8(55):93757–70. doi: 10.18632/oncotarget.20771 (PMC5706833; doi:10.18632/oncotarget.20771)
Supplement: Supplementary file 1 [file oncotarget-08-93757-s001.pdf]

Autophagy protein LC3 regulates the fibrosis of hypertrophic scar by controlling Bcl-xL in dermal fibroblasts

SUPPLEMENTARY MATERIALS

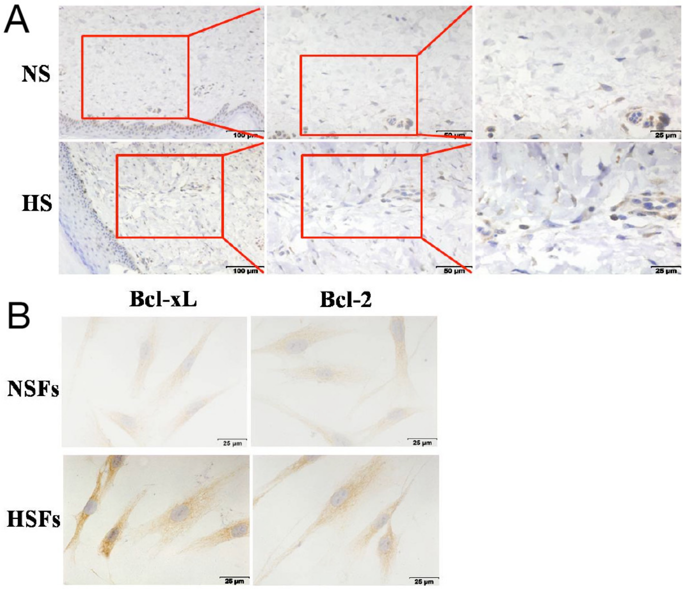

Supplementary Figure 1: Immunostaining analysis of Bcl-xL in HS/HSFs and NS/NSFs. **(A)** Tissues were fixed and prepared samples by immunostaining for observation. Bcl-xL-postive cells in HS were more intensive relative to those in NS. Scale bars, 100 µm, 50 µm and 25 µm. **(B)** NSFs and HSFs were grown on coverslips until they reached 70-80% confluence, fixed in 10% formaldehyde, washed, permeabilized, and blocked. Cells were incubated with a Bcl-xL monoclonal antibody, followed by incubation with a corresponding Streptavidin-peroxidase DAB staining showed that Bcl-xL was localized in HSFs and NSFs. Bcl-xL was distributed in the cytoplasm, with more intensive staining in HSFs than in NSFs. Scale bars, 25 µm.

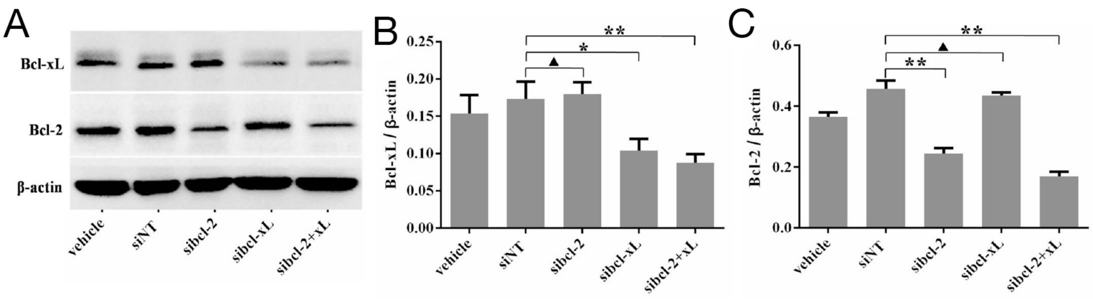

Supplementary Figure 2: The effects of sibcl-xL and sibcl-2 on their protein levels in HSFs. HSFs, with 70-80% confluent, were transfected by siRNAs and their negative control, cultured in DMEM medium for 48 h. **(A)** The expression levels of Bcl-xL and Bcl-2 in silencing for Bcl-xL and Bcl-2 by Western blot analyses. **(B)** Bcl-xL protein expresses and changes in the Bcl-xL/β-actin ratio. **(C)** Bcl-2 protein expresses and changes in the Bcl-2/β-actin ratio. Data are representative of three experiments. n = 3, $^{▲}p > 0.05$, $^{*}p < 0.05$, $^{**}p < 0.01$ compared with the negative control group.
